# Buffalo Academy of Medicine

**Published:** 1904-03

**Authors:** Wm. Irving Thornton


### SOCIETY PROCEEDINGS.

### Buffalo Academy of Medicine

*Section on Medicine, January 12, 1904.*

Reported by WM. IRVING THORNTON, M. D., Secretary.

The regular meeting of the medical section of the Buffalo Academy of Medicine was held at the academy rooms, Public Library Building, Tuesday evening, January 12, 1904. The meeting was called to order by the chairman, Dr. Arthur W. Hurd, at 9 p. m.

Dr. Sidney A. Dunham presented a case which he had treated with the *x*-Ray for epithelioma of the lower eyelid. There was no recurrence one year after treatment.

Dr. LUCIEN HOWE presented the paper of the evening, entitled,

ARE WE AS PHYSICIANS CARRIERS OF CONTAGION?

He believed that laws should be enacted compelling physicians to use proper disinfection of their persons after attending contagious diseases. He recommended that the state bacteriologist determine a standard method of disinfection and that physicians be made liable for malpractice for neglecting the same. A number of instances were cited showing that physicians and surgeons had been the means of spreading contagion.

Dr. H. W. WILLIAMS in discussing the paper referred to Oliver Wendell Holmes's essay on puerperal fever, showing a clear case of infection from the cadaver to the puerperal woman. He believed that we should have considerable more information in regard to the nature of the exanthemata before enacting exact laws. Medical men and students should be instructed in the methods of proper disinfection.

Dr. THOMAS B. CARPENTER spoke of an epidemic of puerperal fever in Buffalo, which compelled a number of physicians to give up their obstetric practice. He said that 1 to 5 per cent. of healthy individuals have the Klebs-Loeffler bacillus in their throats. During an epidemic of diphtheria in a certain town 35 per cent. of the inhabitants had these germs present without any symptoms. In the General Hospital the internes in charge of the contagious ward have the germs almost invariably present. These individuals may convey a virulent form of the disease to a susceptible individual. He did not believe that physicians carried much contagion at present and opposed any legislation in the matter at this time.

Dr. Dunham mentioned an instance where he believed he had carried measles home to his own family.

Dr. J. HENRY DOWD believed that physicians carried contagion and cited an instance. He does not sterilise sounds and never had an infection occur. He simply cleans them with a sterile towel.

Dr. E. E. BLAAUW opposed legislative action in the matter of disinfection.

Dr. E. C. W. O'BRIEN who had charge of Buffalo's small-pox epidemic in 1872, stated that he had come in contact with 1,700 cases and was very sure that he never conveyed the disease to another. In all but six cases the origin of the infection was traced and in no instance could a physician be held responsible. While in the quarantine hospital he kept his hat on and coat buttoned. Before leaving he washed face, hands and moustache in a strong solution of carbolic acid. On leaving the hospital he unbuttoned his coat and did not enter another house for an hour.

Dr. F. THOMA reported conveying measles to a case of scarlet fever and bringing diphtheria to his son after incubating. He did not believe laws are required.

Dr. A. W. HURD stated that the evidence is against much spreading of contagion by physicians and that the enacting of laws as suggested by the essayist would do more harm than good by keeping people from calling physicians in these cases.

In closing Dr. Howe said the point was carried if one individual case could be proven. He believed that young physicians could be found who would devote their entire time to this class of cases.

Meeting adjourned at 10.30 p. m. Members present, 19.

